# Outcome evaluation of prophylactic internal iliac balloon occlusion in the management of patients with placenta accreta spectrum

**DOI:** 10.1186/s42155-024-00466-2

**Published:** 2024-07-23

**Authors:** Asaad Osman, Raj Das, Ana Pinas, Richard Hartopp, Deborah Livermore, Benjamin Hawthorn, Joo-Young Chun, Leto Mailli, Robert Morgan, Lakshmi Ratnam

**Affiliations:** 1 Department of Radiology, St George’s University Hospitals NHS Foundation Trust, Blackshaw Road, London, SW17 0QT UK; 2 Department of Obstetrics and Gynaecology, St George’s Hospital University Hospitals NHS Foundation Trust, Blackshaw Road, London, SW17 0QT UK; 3 Department of Anaesthetics, St George’s Hospital University Hospitals NHS Foundation Trust, Blackshaw Road, London, SW17 0QT UK

**Keywords:** Interventional radiology, Placenta accreta spectrum, Postpartum haemorrhage, Prophylactic occlusion balloon, Uterine artery embolisation, Vascular complication

## Abstract

**Purpose:**

To evaluate outcomes and complications of prophylactic internal iliac balloon occlusion (PIIBO) in the management of patients with placenta accreta spectrum (PAS) at a large regional referral centre.

**Materials and methods:**

A retrospective review of all PIIBO for PAS performed over a 12-year period (2010–2022). Information for analysis was gathered from the local RIS/PACS and clinical documentation. Collected data included patient demographics, indication for procedure, sheath insertion and removal time, total duration of balloon inflation and complications that occurred.

**Results:**

106 patients underwent temporary internal iliac artery balloon occlusion within the 12-year period. All procedures utilised bilateral common femoral artery punctures, 6Fr sheath and 5Fr Le Maitre occlusion balloons. Catheters were successfully positioned and balloons inflated in obstetric theatre following caesarean delivery in 100% of the cases. The uterus was conserved in every case. There was no maternal mortality or foetal morbidity.

Twenty patients (18.9%) had some form of complication that required further intervention. Of these, 7(6.6%) had post-operative PPH, which was treated with uterine artery embolisation; and 13 (12.3%) had arterial thrombus which required aspiration thrombectomy. All procedures were technically successful with no long-term sequelae.

**Conclusion:**

PIIBO plays an important part in reducing morbidity and mortality in patients with PAS. Clear pathways and multidisciplinary team working is critical in the management of these patients to ensure that any complications are dealt with promptly to avoid long-term sequelae.

## Introduction

Placenta accreta spectrum (PAS) is subdivided into three subtypes in order of severity with risk of maternal haemorrhage and death; accreta (superficial invasion of the placental villi into the myometrium), increta (deep invasion of the myometrium) and percreta (invasion of the serosa with or without extension into the periuterine tissues) [1–3].

The incidence of PAS has increased from 1.7 per 10,000 births to 577 per 10,000 births largely due to an increased rate of caesarean delivery and increasing maternal age [4, 5]. Early diagnosis in high-risk patients allows treatment at a tertiary centre by an experienced multidisciplinary team and is associated with improved maternal and neonatal outcomes [6].

Traditionally, due to the high risk of catastrophic haemorrhage, PAS has been managed by planned delivery via caesarean hysterectomy [7, 8]. However, this is associated with significant maternal morbidity with mortality rates as high as 7% [9]. Many women also experience negative psychological effects after hysterectomy related to the loss of fertility and the impact on a woman’s societal status and self-esteem [10].

Adjunctive interventional radiology (IR) techniques include prophylactic occlusion balloon placement and uterine artery embolisation (UAE) in cases of uncontrolled haemorrhage [2, 11, 12].

This retrospective study aims to evaluate the outcomes and complication rates from our experience as a large regional tertiary referral centre utilising prophylactic internal iliac balloon occlusions (PIIBO) for the management of patients with PAS. We also describe how the patient management and procedural technique has been adapted to mitigate complications in this complex patient group.

## Materials and methods

Retrospective evaluation was carried out of all PIIBO for PAS performed over a 12-year period (January 2010—January 2022). Patients were identified with PAS based on antenatal ultrasound identified placental invasion. Data was collected from 106 patients records and included patient demographics, indications of procedure (type of PAS), sheath and balloon insertion and removal time, total duration of balloon inflation, estimated blood loss (EBL), and complications that occurred. The data includes the first 37 patients in the series that were previously published from our institution from 2010 – 2016 [13].

### Procedural steps Fig. 1

**Fig. 1:**
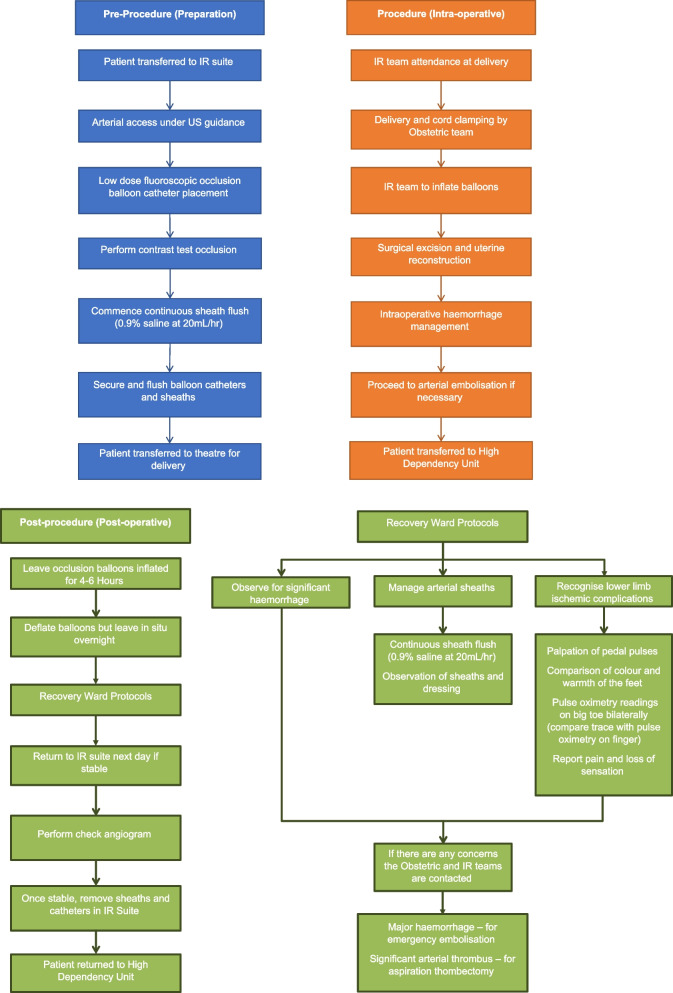
Flow chart of procedural steps

The patient is initially transferred to the IR suite for the procedure. Arterial access to both common femoral arteries is gained under ultrasound guidance. No heparin is administered at the time of sheath placement. Occlusion balloon catheters (5Fr embolectomy catheters, LeMaitre Vascular, Burlington, USA) are placed with the catheter tips positioned into the anterior division of the contralateral internal iliac artery. Radiation exposure is minimised using pulsed low dose fluoroscopic guidance at 2 pulses per second [14] Fig. 1.

**Fig. 2:**
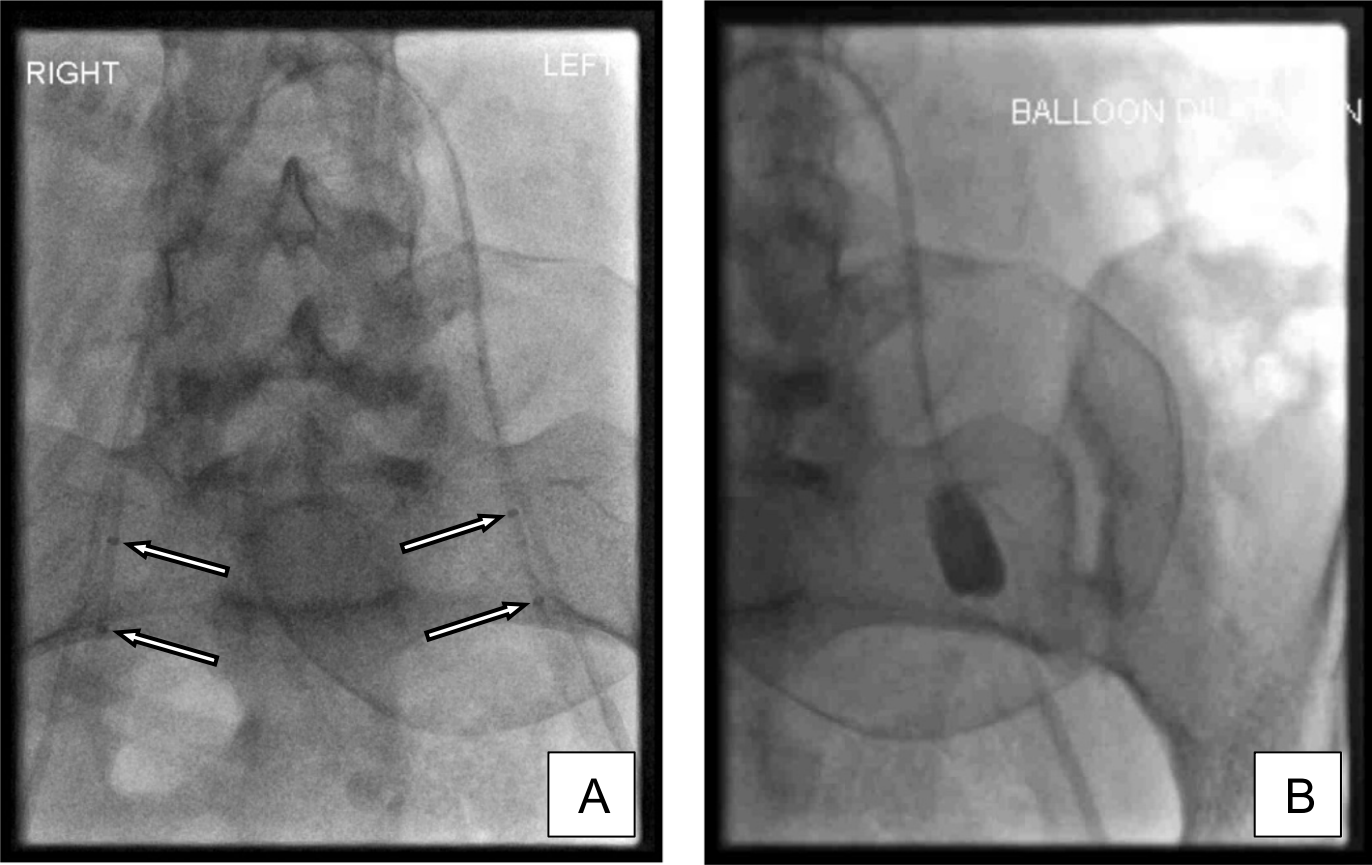
**A** Occlusion balloon catheters with their tips positioned within the anterior division of the contralateral internal iliac artery. (Arrows demonstrating location of balloon markers). **B** Test inflation of occlusion balloon catheter at time of insertion

Contrast used for a brief test occlusion is recorded and left in the syringe attached to each balloon catheter. Volume required for balloon inflation to achieve occlusion varies between 1-2 ml and is assessed fluoroscopically for all patients. Once inserted, each sheath is flushed continuously with a bag of 500 ml normal saline 0.9% running at an infusion rate of 20 ml/hr via a pump. Balloon catheters and the sheaths are sutured and dressed to minimise the risk of dislodgement during patient transfer to the obstetric theatre Fig. 2.

The IR team attend the delivery to ensure correct balloon inflation and are prepared to proceed with immediate embolisation if there is uncontrolled haemorrhage. After delivery and clamping of the umbilical cord, each balloon is inflated and the surgery is completed. The obstetrician surgically excises as much of the placenta as possible along with any myometrium and reconstructs the uterus [10]. If there is no significant intra-operative haemorrhage, the balloon catheters are left inflated for 4–6 h, then deflated but left in situ overnight. If the patient remains stable, an angiogram is performed, and the sheaths and balloon catheters are removed in the IR suite the next morning.

**Fig. 3:**
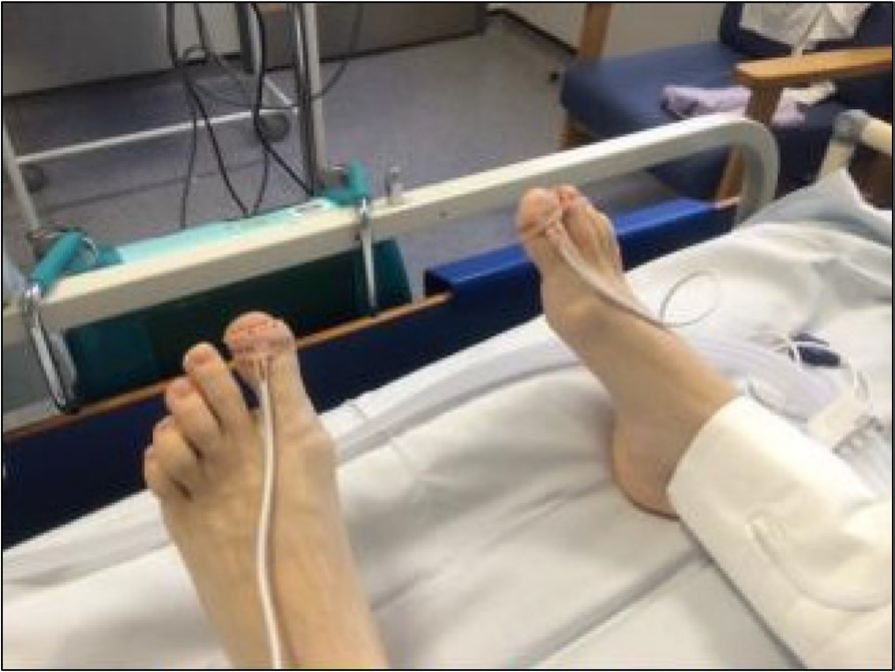
Pulse oximetry probes placed on patient’s feet which are evaluated every 15 min during recovery period to assess perfusion

Multidisciplinary protocols are in place to ensure that staff on the recovery wards are familiar with the management of arterial sheaths, recognition of lower limb ischaemic complications and observations for significant haemorrhage Fig. 3.

If significant intraoperative haemorrhage occurs despite inflation of the occlusion balloons, the presence of intra-arterial catheters in the internal iliac arteries allows for rapid progression to arterial embolisation [15].

### Clinical outcomes and statistical analysis

Primary outcomes evaluated were EBL and total arterial sheath indwelling time for the different grades of PAS. These have been reviewed in two groups; those without complications (non-complicated group 1) and those with complications (complicated—group 2). Complications were graded according to the Cardiovascular and Interventional Radiological Society of Europe (CIRSE) classification [16].

For statistical analysis, independent-samples *t* test, chi-squared test and Mann–Whitney *U* test used to evaluate means and Kruskal–Wallis test used to evaluate more than two variables. A *P* value < 0.05 was considered significant. Statistical analysis was performed with GraphPad Prism version 9.5.1 (GraphPad Software, Inc, La Jolla, CA, USA).

## Results

A total of 106 patients underwent PIIBO in the 12-year period, with a mean age of 36 years (range 24–51). PAS presented as: 61 (57.5%) accreta, 7(6.6%) increta, and 38 (35.8%) percreta. Patient demographics are listed in Table 1.

**Table 1:** Patient demographics

		Result
Age	Mean	35.8
	Median	36
	Range	24–51
Diagnosis	Accreta	61 (57.5%)
	Increta	7 (6.6%)
	Percreta	38 (35.8%)
Estimated Blood Loss	Mean	2907.2
	Median	2000
	Range	400–17000
Sheath Time (minutes) (Days:Hours:Minutes)	Mean	1305 (0:21:45)
	Median	1402 (0:23:22)
	Range	330–1851 (0:5:30–1:6:51)

All procedures were technically successful with no long-term sequelae identified, with the uterus being conserved in every case and no recorded maternal mortality or foetal morbidity.

There was no statistical difference in mean EBL and total sheath indwelling time in the three PAS subgroups. The overall total mean EBL was 2907 mL. The mean EBL after the procedure was 2687 mL, 3100 mL and 3281 mL, and the total sheath time was 1333, 1083 and 1352 min for the accreta, increta and percreta groups respectively. Comparisons of different grades of PAS are listed in Table 2.

**Table 2:** Comparison of different grades of MAP

		Accreta	Increta	Percreta	*P* value
Number of patients		61	7	38	
Total Sheath Time (minutes) (Days:Hours:Minutes)	Mean	1333 (0:22:13)	1083 (0:18:03)	1352 (0:22:32)	Accreta vs Increta vs Percreta 0.3082
	Median	1405 (0:23:25)	1366 (0:22:46)	1404 (0:23:24)	
	Range	358–1851 (0:5:58–1:6:51)	422–1489 (0:7:02–1:0:49)	338–1853 (0:5:38–1:6:53)	
	Mean CI 95%	1254–1413 (0:20:54–0:23:33)	678–1488 (0:11:18–1:0:48)	1269–1434 (0:21:9–0:23:54)	
Estimated Blood Loss (mL)	Mean	2687	3100	3281	Accreta vs Increta 0.5532
	Median	1800	3100	2100	Accreta vs Percreta 0.3690
	Range	400–10000	1500–5100	1000–17000	Increta vs Percreta > 0.9999
	Mean CI 95%	2146–3228	2014–4186	2351–4211	Accreta vs Increta vs Percreta 0.1784

Table 3 lists the complications. A total of 29/106 (27.4%) patients had a complication. Of those, 9/106 (8.5%) did not require additional therapy (CIRSE grade 1), 8/106 (7.5%) had arterial thrombus formation and 1/106 (0.9%) had an arterial dissection. 20/106 (18.9%) required further intervention (CIRSE grade 2/3) with no long-term consequence. A total of seven patients required UAE for uncontrolled postpartum haemorrhage (PPH); three accreta, two increta, and two within the percreta subgroups, with average time to UAE following balloon deflation being 130 min in all groups. Analysis of the three groups found that the degree of placental invasion was not a significant predictor for requirement of definitive embolization. The chi-square statistic is 5.8688 with a *p*-value of > 0.05.

**Table 3:** Complications

		Accreta	Increta	Percreta	Total
Uterine Artery Embolisation		3	2	2	7 (6.6%)
Average Time to UAE (minutes) (Hours:Minutes)		178 2:58	83 1:23	55 0:55	130 2:10
Thrombosis	Total	15	2	4	21 (19.8%)
	Significant	7	2	4	13 (12.3%)
Dissection	Total	1	0	0	1 (0.9%)
	Significant	0	0	0	0 (0%)

**Fig. 4:**
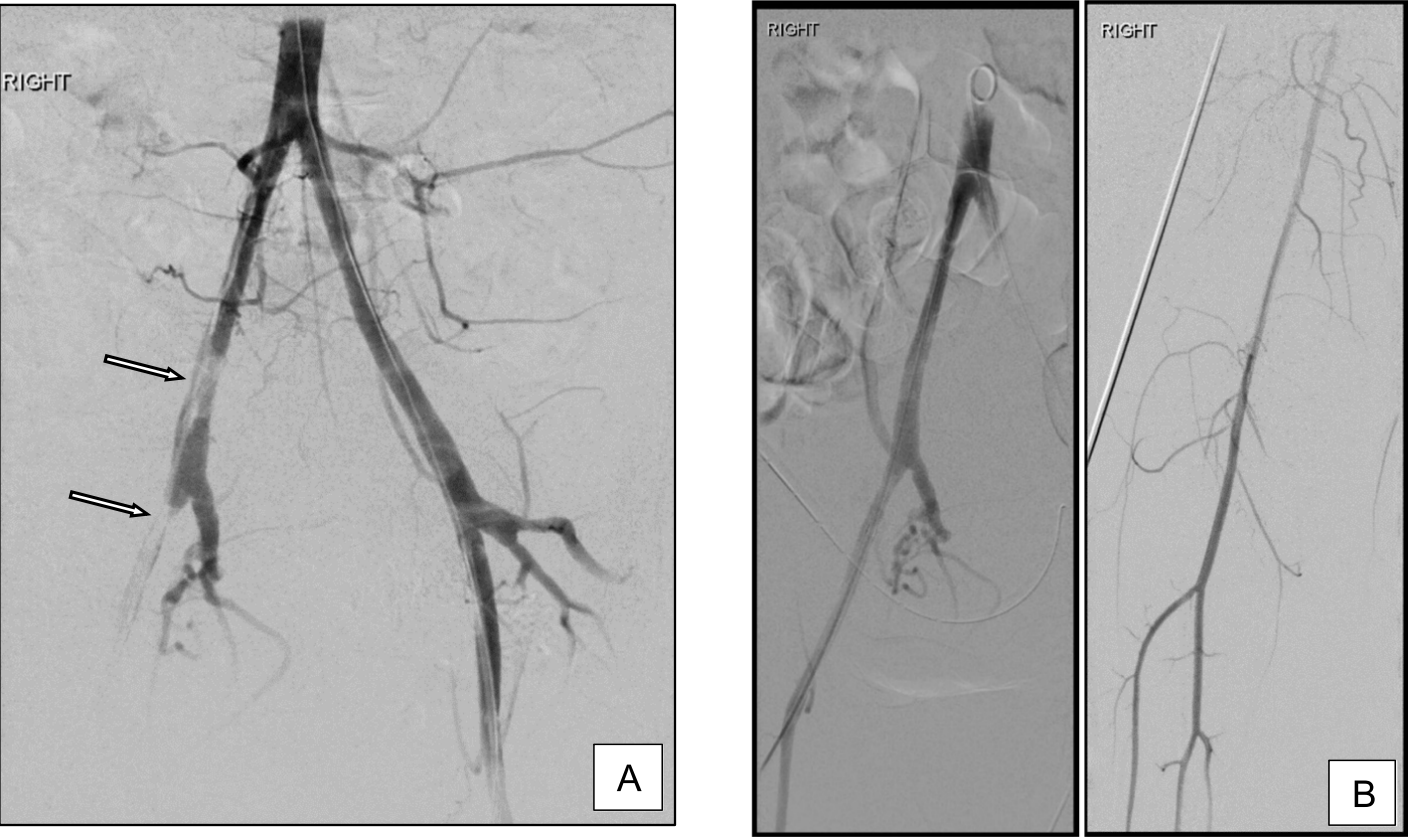
**A** Angiography prior to sheath removal demonstrates thrombus within right common iliac and external iliac arteries (arrows). Patient remained asymptomatic with warm, well-perfused leg throughout. **B** Patent arteries following aspiration thrombectomy. Run off vessels all intact. Leg monitored continuously and duplex ultrasound subsequently confirmed resolution of thrombus. No adverse sequelae

21 (19.8%) patients developed arterial thrombosis, eight of those developed partial, non-flow limiting asymptomatic thrombus within the internal iliac or common iliac arteries not requiring any further management. 13 (12.3%) patients had arterial thrombus requiring further intervention (seven accreta, two increta and four percreta subgroups). These had thrombus within the external iliac or common femoral arteries and all underwent successful aspiration thrombectomy (Fig. 4). Aspiration was performed mostly with simple suction on a catheter or using a variety of aspiration catheters available. Choice of suction catheter varied with operators. One patient had asymptomatic distal embolisation of thrombus resulting in tibioperoneal trunk occlusion. This was managed conservatively with therapeutic subcutaneous heparin and completely resolved on pre-discharge Doppler ultrasound. Two of the patients requiring thromboaspiration described paraesthesia and a cold foot in the immediate post-operative setting; the remainder were asymptomatic. Vascular surgical consultation was carried out in all cases with residual thrombus, but no surgical intervention was required in any of our cases.

One asymptomatic patient had a non-flow limiting focal dissection within the right external iliac artery, thought to be secondary to sheath insertion, which was treated conservatively. There were no femoral access site or closure device related complications.

Comparison of complications are summarised in Table 4. The mean EBL was significantly higher in Group 2 patients with complications (4594 mL) compared to those with Group 1 patients with no complications (2596 mL) (*P* = 0.0128). Only the UAE group had significantly higher EBL when compared to the non-complicated group (*P* = 0.0010). There was no statistically significant correlation between non-complicated EBL and thrombosis rate (*P* = 0.5132). Furthermore, no statistically significant association was found when comparing EBL in the each of the subgroups of complications (e.g. UAE group (7171 mL) vs thrombosis group (4089 mL) (*P* = 0.5000)).

**Table 4:** Comparison of non-complicated (Group 1) vs complicated patients (Group 2)

		Non-complicated	Complicated			*P* value
**Total**		86 (81.1%)	Total	UAE	Thrombosis	
			20 (18.9%)	7 (6.6%)	13 (12.3%)	
**Estimated Blood Loss** **(mL)**	Mean	2596	4594	7171	4089	**Non-Complicated vs Complicated 0.0128**
						**Non-complicated vs UAE** **0.0010**
	Median	1900	3900	6000	3800	Non-complicated vs Thrombosis 0.5132
	Range	400–8000	1200–17000	4000–17000	1200–10000	Complicated vs UAE 0.4170
						Complicated vs Thrombosis > 0.9999
	Mean CI 95%	2214–2979	2472–6716	3085–11258	1932–6246	UAE vs Thrombosis 0.5000
**Total Sheath Time (minutes)**	Mean	1305	1279	1507	1279	Non-complicated vs Complicated 0.7183
	Median	1390	1439	1551	1439	Non-complicated vs Thrombosis > 0.9999
	Range	330 – 1851	427–1592	1377–1592	427–1565	Non-complicated vs UAE 0.9836
						Complicated vs UAE & Thrombosis > 0.9999
	Mean CI 95%	1293–1394	882–1391	1223–1790	962–1592	UAE vs Thrombosis 0.7716

Analysis of the arterial sheath indwelling time in group 1 and group 2 and between the requirement for UAE and significant thrombosis showed no correlation between different variables.

## Discussion

The use of occlusion balloons in patients with PAS has been reported to improve patient outcomes. A recent systematic review of 19 studies reported a wide variability in outcomes, concluding that available data for the efficacy of prophylactic occlusion balloon catheter (POBC) in cases of planned caesarean hysterectomy for known PAS provides limited evidence that these techniques are beneficial in reducing blood loss, but can have significant complications [17].

A prospective observational study of 23 patients with PAS treated with caesarean hysterectomy and 30 patients treated with pre-operative balloon catheter insertion demonstrated a significant reduction in EBL and transfused blood products between the two groups but only in patients with placenta percreta [18]. The Californian registry data (2020) compared outcomes of 28 patients with PAS who underwent caesarean hysterectomy with aortic/internal iliac artery balloon occlusion, with 18 patients who underwent surgical ligation of internal iliac arteries and 125 patients who had no adjunctive procedures. The aortic/internal iliac artery balloon occlusion group had significantly lower EBL (30.9% decrease, *P* < 0.001), transfusion requirements, intensive care unit admission and adverse events when compared to the other two groups [19].

These findings were mirrored in our early experiences of 37 patients who underwent PIIBO for PAS as an adjunct to conservative surgery from 2010–2016 and have been incorporated in our current review [13]. A more recent study in 90 patients with PAS also showed a significant decrease in EBL and transfusion volume with 9% complication rate in the POBC group (*n* = 29) [11].

On the other hand, some studies have shown no benefit, with reports of high EBL and high rates of hysterectomy despite occlusion balloon use. Salim et al. conducted a prospective randomised trial which compared the use of POBC in 13 patients with a 14-patient control group without balloon catheters. No difference was shown between the two groups comparing the number of women with blood loss > 2500 ml, transfusion requirements, duration of surgery, peri-partum complications and length of hospital stay. However, the sample size was small and not all cases had histological confirmation of PAS [20].

Shrivastava et al. presented retrospective results of 69 patients concluding that iliac POBC does not result in reduced blood loss or transfusion requirements. However, POBC were used in only 28% of the patients studied and results were not categorised by depth of placental invasion, both confounding factors which may limit the ability to detect a significant treatment effect. In addition, 16% of women with POBC developed balloon catheter related complications. Complications reported include internal iliac artery dissection and occlusion requiring iliofemoral bypass surgery [21] and popliteal artery thrombus after internal iliac POBC without any sequelae [22].

Our results showed a mean EBL of 2907 mL (Table 1) which is significantly lower than mean EBL for hysterectomies which are reported as averaging 5300 mL [13]. We found no significant link between the complication rate and type of PAS, total sheath indwelling time, or EBL. However, there is a significant relationship between the volume of EBL and rate of complications, specifically patients who subsequently had UAE (*P* = 0.0010) (Table 4).

The most common complication encountered was arterial thrombosis. No specific cause for this has been identified on further analysis. No correlation was found between the length of sheath indwelling time with the development of arterial thrombosis, however, of note, the sheath indwelling time across all the patient groups were similar. Our complication rates are comparable to those in already published case series. However, when compared to our prior institution results there has been an overall increase in the significant thrombosis rate from 8% to 12.3*%*. 8 patients had thrombus within internal iliac arteries which have been noted as complications, but these are not treated and do not cause any concern given our experience in occluding internal iliac arteries prophylactically in the management of aortic aneurysms [23]. We hypothesize that the increased bleeding in these cases with the resultant increase in blood product requirements is likely to have caused these patients to become more coagulopathic with a resultant increase in thrombosis rate.

Complications are prevented by high levels of vigilance in all staff caring for these patients. Any clinical signs of impaired perfusion are dealt with immediate return to the IR suite for angiography, and aspiration thrombectomy if required. Paraesthesia and a drop in the pulse oximetry reading from the affected limb were noted in two patients developing significant thrombosis. Thus, pulse oximetry monitoring is part of the post procedural observation protocol. The remaining patients all maintained unchanged oximetry readings in their feet which remained warm and well perfused. This suggests that despite the level of thrombus visualised angiographically, the degree of thrombosis was not clinically significant and may have resolved spontaneously without treatment. However, given that we had arterial access, in all cases where the thrombus was accessible and deemed to be of concern due to the location and volume, aspiration was successfully performed.

In the one case where the thrombus was not accessible, heparinisation was commenced as soon as deemed safe following multidisciplinary input. This will depend on the level of concern for bleeding on a case-by-case basis. Our normal practice is to commence prophylactic heparin injections 12 h following caesarean section. All patients found to have thrombus had duplex ultrasounds prior to discharge and all were found to be clear of thrombus.

This study is limited by its retrospective design, the significant variation in degree of placenta percreta in the more complex cases and the variability in procedural approach between different surgeons which may influence outcomes.

In response to our experience over 12 years and the thrombotic complications seen, we have introduced a number of specific procedural changes as below in addition to the clinical observations described above which were instituted early in our experience.

- We do not have access to a hybrid theatre. Patients were previously transferred to the IR suite for balloon placement, then moved to obstetric theatres. The acquisition of a high-quality mobile fluoroscopy unit combined with our experience in placing the balloons has enabled a shift to the entire procedure taking place in obstetric theatres resulting in shorter procedural times, improved patient pathway and reduced radiation as no need to reconfirm balloon position prior to commencing the caesarean section.
- Previously balloon catheters were deflated but left in situ within sheaths overnight should the need arise to carry out embolisation for ongoing PPH. Analysis of our results demonstrated that of the 7 cases requiring UAE, a decision to proceed to embolisation was made within 4 h of the end of the procedure. Together with the thrombotic complications and the commencement of extended working days in Interventional Radiology to 8 pm, this led to a decision to remove balloons and sheaths on the evening of the same day.
- Regular MDT review of planned cases and review of any complications or difficulties in cases performed led by obstetrics and attended by foetal medicine practitioners, anaesthetists, midwives and interventional radiologists with sharing of learning and cascade to the rest of the team.

## Conclusion

In our experience, PIIBO is an effective option in reducing morbidly and mortality in patients with PAS if used selectively with strict protocols in place. This study highlights the relationship between EBL and arterial thromboses which should lead to increase vigilance in these patients. Changes in practice have been introduced to streamline management and mitigate known complications. We have identified that the full engagement of the multidisciplinary teams with clear pathways is critical in managing these complex patients to ensure maximal benefit whilst dealing with complications promptly to avoid any long-term sequelae.

## Data Availability

The datasets used and/or analysed during the current study are available from the corresponding author on reasonable request.
